# Factors affecting compliance in the use of prescription footwear in the management of diabetic foot disease

**DOI:** 10.1186/1757-1146-3-S1-P18

**Published:** 2010-12-20

**Authors:** Bryan Watters

**Affiliations:** 1 Cwm Taf Health Board, Rhondda Cynon Taf, UK

## Introduction

A poster presentation will be prepared to disseminate the result of research conducted to investigate the potential for the Health Belief Model as a tool to understand the factors affecting compliance with the use of prescribed footwear in diabetic foot disease.

## Research design and methods

A self-completion postal questionnaire was developed and applied in a South Wales Valley community to investigate the health beliefs of 72 individuals provided with prescription footwear for advanced diabetic foot disease and to investigate the impact of these beliefs on their compliance with prescription footwear.

## Results

46.6% of respondents were found to be compliant with their use of prescribed footwear at the 9.6 hours per day threshold. Prior history of ulceration was found to be associated with increased likelihood of compliance (ϕ= 0.286, p= 0.031), which contests the finding of McFarlane & Jensen(2003). Recollection of advice to wear the prescribed footwear indoors was also significantly associated with compliance (ϕ = - 0.486, p<0.01). Patients who perceived prescribed footwear as beneficial used them for a greater number of hours per day (r=0.286, p =0.025). Patients who perceived diabetic foot ulceration as serious were more likely to perceive prescribed footwear as beneficial (r=0.349, p=0.005) and patients who perceived prescribed footwear as beneficial were less likely to rate barriers to compliance as high (r= -0.262, p=0.038). The correlation between the summary health belief scale and reported daily hours of prescription footwear was not significant (r=0.184, p=0.163). An improved health belief model has been constructed to enhance understanding of the factors affecting compliance in this context.

It is recommended that the NHS provision of prescription footwear should be focused toward patients with a previous history of ulceration and those for whom ulceration is imminent. Patients with pre-ulcerative diabetic foot disease should be counseled to wear protective commercial footwear indoors as well as when walking outside.

